# The effect of intraoperative specimen inking on lumpectomy re-excision rates

**DOI:** 10.1186/1477-7819-8-4

**Published:** 2010-01-18

**Authors:** Mansher Singh, Gayatri Singh, Kevin T Hogan, Kristen A Atkins, Anneke T Schroen

**Affiliations:** 1 Department of Surgery, University of Virginia, Charlottesville, Virginia, 22908, USA; 2 Department of Surgery, All India Institute of Medical Sciences, Ansari Nagar, New Delhi, 110029, India; 3 Department of Pathology, University of Virginia, Charlottesville, Virginia, 22908, USA

## Abstract

**Background:**

Lumpectomy re-excision to obtain negative margins is common. We compare the effect of two specimen orientation approaches on lumpectomy re-excision rates.

**Methods:**

All women undergoing lumpectomy for breast cancer by a single surgeon between 03/2007 - 02/2009 were included. Lumpectomies underwent standard inking (SI) after surgery by a pathologist from 03/2007-02/2008 while intraoperative inking (II) with direct surgeon input was done from 03/2008-02/2009. Rates of margin positivity and re-excision were compared between these methods.

**Results:**

65 patients were evaluated, reflecting SI in 39 and II in 26 cases. Margin positivity rates of 46% [SI] vs. 23% [II] (p = 0.06) and re-excision rates of 38% [SI] vs. 19% [II] were observed. Residual disease at re-excision was found in 27% [SI] vs. 67% [II] of cases.

**Conclusions:**

Intraoperative inking in this practice offered a simple way to reduce re-excision rates after lumpectomy and affect an improvement in quality of patient care.

## Background

Achieving negative margins remains one of the most important determinants for local recurrence following breast conserving therapy [1]. Re-excision rates after lumpectomy for the treatment of breast cancer to achieve negative margins have been reported between 20-60% [2-5]. Re-excision lumpectomy may lead to diminished cosmetic results, delays in adjuvant therapy, and additional anxiety and expense. In order to minimize the tissue volume removed at re-excision, directed re-excision can be performed with accurate specimen orientation [6]. Directed re-excision of positive margins typically relies on the use of up to six multi-colored inks and reporting of separate margin status or widths. Traditionally, the contour of the lumpectomy specimen is oriented by the surgeon by placing stitches to mark two or more of the six sides which later allows the pathologist to reorient the specimen and ink it with six different colors to mark the anterior, posterior, medial, lateral, superior and inferior sides. Discordance between the surgeon and the pathologist in margin orientation would influence the accuracy of re-excisions. A discordance rate of 31% has recently been reported [7]. In a quality improvement effort within our practice, we hypothesized that re-excision rates after lumpectomy can be reduced and the accuracy of margin re-excisions increased through direct surgeon involvement in specimen inking in the operating room.

## Methods

A retrospective study was performed comparing lumpectomy re-excision rates using two different specimen orientation methods. All consecutive female patients undergoing lumpectomy for a known cancer diagnosis by a single surgeon between March 2007 and February 2009 were included. Charts from patients with both known invasive and/or ductal carcinoma in situ (DCIS) were reviewed. Patients undergoing lumpectomy after neoadjuvant therapy were excluded. Lumpectomy specimens retrieved between March 2007 - February 2008 were oriented by the surgeon with three sutures indicating the lateral, superior, and anterior sides of the lumpectomy. The specimens were inked by pathology at a later time in the laboratory with six separate colors. This is referred to as the standard inking (SI) regimen. Lumpectomy specimens retrieved between March 2008 - February 2009 were similarly marked with three sutures and then inked in the operating room by the surgeon and pathologist together using the same six colors. This is referred to as the intraoperative inking (II) regimen. Specimens removed with wire localization in the SI group were first sent to mammography for specimen radiograph prior to being sent to pathology for inking; in the II group, specimens were inked prior to being sent to mammography. Specimen radiographs were performed for all lumpectomies with wire localization but compression was not routinely used during specimen radiography. Lumpectomies performed at a freestanding outpatient surgery center were included in the SI group as intraoperative inking by pathology was not available at this facility. Additional margins were excised at time of original lumpectomy at the surgeon's discretion based on gross or radiographic impression of a close margin.

Patient records were reviewed for patient age, wire localization, tumor size, tumor histology, margin status, additional margins removed at original surgery, lumpectomy resection volume, and estrogen receptor (ER) status. A positive margin was defined as presence of invasive or intraductal cancer at the inked margin. A close margin was defined as presence of DCIS within 3 mm of the inked margin since it is our institutional practice to re-excise these close margins. Cases with positive and close margin(s) were reviewed for margin re-excision and whether residual disease was identified at re-excision. The correlation between margin status and various patient and tumor characteristics was evaluated. Analysis was performed using Microsoft Excel and Graph Pad Prism software. A two-tailed chi-square test was used to compare proportions while the Student's t-test was used to compare means. A p-value of 0.05 was considered statistically significant. This study was approved by the University of Virginia Institutional Review Board.

## Results

Sixty-five consecutive, female patients underwent primary lumpectomy for a known cancer diagnosis over the 24-month duration of the study. The SI cohort included 39 patients and the II cohort included 26 patients. A general comparison between the two groups is shown in Table 1. The groups appear similar in many patient and tumor characteristics. However, the average tumor size was larger in the II group; consequently the proportion of lumpectomies performed for palpable tumors and the average volume of the original lumpectomies was larger in the II group. One particularly large lumpectomy was performed in this group. This case involved a central lumpectomy where the transverse diameter of the elliptical incision was larger than required to achieve negative margins but would afford a more cosmetic closure. When comparing only lumpectomies with wire localization between the two groups, however, the average lumpectomy volume was more closely matched with 54.72 cm^3 ^for the II group and 45.16 cm^3 ^in the SI group.

**Table 1:** General Characteristics for 34 Consecutive Breast Cancer Patients Treated with Breast Conserving Surgery

	Specimen Inking Regimen		
	
Patient, Tumor, or Surgery Characteristic	Intraoperative (n = 26)	Standard (n = 39)	p-value
Patient age, mean yrs (range)	64 (41-93)	61 (41-84)	0.59
			
Pre-operative histological diagnosis - N (%)			
DCIS alone	3 (12%)	12 (31%)	
Invasive ductal carcinoma	17 (65%)	18 (46%)	
Invasive ductal carcinoma + DCIS	5 (19%)	5 (13%)	
Invasive lobular carcinoma (+/- DCIS)	1 (%)	4 (10%)	0.18
			
Tumor grade - N (%)			
Grade 1	3 (12%)	14 (36%)	
Grade 2	11 (42%)	16 (41%)	
Grade 3	12 (46%)	9 (23%)	0.04
			
ER positive - N (%)	20 (77%)	34 (87%)	0.28
			
Extensive intraductal component - N (%)	3 (12%)	4 (10%)	0.87
			
Type of surgery - N (%)			
Lumpectomy with wire localization	17 (65%)	35 (90%)	
Lumpectomy	9 (35%)	4 (10%)	0.03
			
Invasive tumor size at excision - mean cm (range)	1.58 (0.4-4.3)	1.16 (0.3-2.9)	0.24
			
Original lumpectomy volume - mean cm^3 ^(range)	73.2 (3.5-206.7)	47.0 (6.3-139.1)	0.08
			
Patients in whom extra margins were excised at original surgery - N (%)	18 (69%)	28 (72%)	0.82
			
Number of extra margins excised per patient at original surgery - mean (range)	2.0 (0-6)	2.3 (0-6)	0.91

The frequency of positive or close margins and results at re-excision are shown in Table 2. Positive/close margins were found in 46% (n = 18) of cases in the SI group compared to 23% (n = 6) of the II group, a relative reduction of 50% (p = 0.06). In both groups, some patients declined re-excision. Of 6 patients with positive/close margins in the II group, five patients underwent further surgery with 3 patients having a re-excision lumpectomy. Of 18 patients with positive/close margins in the SI group, fifteen patients underwent further surgery with 11 having a re-excision lumpectomy. A 50% reduction in the actual re-excision rate was seen in the II group (19%) as compared to the SI group (38%). Residual disease was present in 2 out of 3 patients (67%) from the II group at re-excision as compared to 3 out of 11 patients (27%) in the SI group. This corresponded to residual disease being present in 4 of 5 specific margins (80%) in the II group at re-excision compared to 4 of 20 (20%) margins in the SI group. It should be noted, however, that the extent of DCIS in two of the II patients made positive re-excisions more likely.

**Table 2:** Comparison of Intraoperative Inking Regimen with Standard Inking Regimen

	Specimen inking regimen		
	
Measurement	Intraoperative (n = 26)	Standard (n = 39)	P-value
1a. No. patients with positive/close margins at original surgery	6 (23%)	18 (46%)	0.06
*Positive margins	4 (15%)	7 (18%)	
*Close margins	4 (15%)	15 (38%)	
			
1b. No. patients who underwent re-excision	5 (19%)	15 (38%)	0.16
†Re-excision lumpectomy	3 (12%)	11 (28%)	
†Mastectomy	3 (12%)	6 (15%)	
			
2. No. patients with residual disease identified on re-excision lumpectomy	2 of 3 (67%)	3 of 11 (27%)	0.5
			
3. Residual disease identified in the specific margins at re-excision lumpectomy	4 of 5 (80%)	4 of 20 (20%)	0.02
			
4a. No. patients with positive/close margins on original lumpectomy specimen alone	9 (35%)	22 (56%)	0.08
			
4b. No. patients with positive/close margins on original lumpectomy specimen who had extra margins taken at original surgery	5 of 9 (55%)	11 of 22 (50%)	0.78
			
4c. No. patients with positive/close margins on original lumpectomy specimen whose margins were cleared by extra margins taken at original surgery	3 of 9 (33%)	4 of 22 (18%)	0.36
			
5. No. extra margins taken at original surgery that corresponded to positive/close margins on original lumpectomy	6 of 7 (86%)	15 of 19 (79%)	0.70

* Some patients had positive as well as close margins

† Some patients first underwent re-excision lumpectomy prior to ultimately converting to mastectomy

To assure that technical aspects of the surgery were performed similarly between the two groups, we examined concordance of extra margins excised at original lumpectomy and rate of these margins corresponding to positive/close margins on the actual lumpectomy specimen. As shown in Table 2, no significant differences were identified between the SI and II groups in concordance of extra margins taken at original lumpectomy. Additionally, the relationship between various patient and tumor characteristics and margin status was evaluated. No association between margins status at original excision and patient age, ER status, invasive tumor size, or lumpectomy volume was identified. However, cases with positive margins appeared more likely to involve DCIS than those with negative margins at first surgery (88% vs. 68% respectively, p = .08) and much more likely to involve an extensive intraductal component (25% vs. 2% respectively, p = .008).

## Discussion

A relatively simple change in our lumpectomy specimen inking practice produced a measurable improvement in quality of patient care by reducing re-excision rates by 50%. Intraoperative inking presents one of several ways that a surgeon's involvement in margin assessment can help reduce re-excision rates. Other examples include use of intraoperative ultrasound or use of cavity shaving [6,8]. The simplicity, low cost, and no additional training make intraoperative inking an easy tool to apply in most settings. The method capitalizes on the surgeon's unique ability to conceptualize the lumpectomy cavity and recreate more accurate margin definitions on the specimen.

Our study was not designed to specifically explain why intraoperative inking may be superior to standard inking in reducing re-excision rates; rather the study's purpose was to demonstrate whether an individual surgeon's practice could be improved by this change in specimen handling. A possible explanation includes intraoperative inking improving margin concordance and accuracy. Alternately, intraoperative inking may benefit from less ink bleeding or changes in margin measurements resulting from structural changes to the tissue over time, such as flattening of the specimen or fat retraction. Additional limitations of this study may include generalizability of the results given that the study reflects the experiences of a single surgeon and institution. A potential bias could have been introduced if more additional new margins had been excised at the original surgery in the II group than in the SI group. However, additional margins were taken slightly more often and in somewhat greater number per case in the SI group. Finally, the larger resection volume of original lumpectomy was higher in the II group. This may reflect the larger tumor size seen in this group. Prior work has shown that smaller lumpectomy volumes result in higher re-excision rates and that palpability of tumors functions as a predictor of negative margins [9]. Lumpectomy volumes for non-palpable lesions were similar in our study, suggesting that no major change in surgical technique was employed. Furthermore, the lumpectomy volumes and invasive tumor sizes were equivalent between cases with positive and negative margin status. Lastly, a greater proportion of patients with DCIS or lobular cancer, both of which have been associated with greater positive margin rates, were found in the SI group [4,9]. A larger study would be valuable in validating our results between groups of equal resection volumes and tumor histology distribution, and across more practice settings.

## Conclusions

Intraoperative inking of lumpectomy specimens is a cost effective, simple method which can reduce the need for lumpectomy re-excision and improve accuracy of directed margin re-excisions. This technique can be readily applied in many settings, whether performed by the surgeon and the pathologist together or the surgeon alone. Although this is a small study, these provocative results prompted a simple change in practice at our institution to attain improvements in quality of patient care by reducing the number of operations required to deliver breast conserving surgery.

## Competing interests

The authors declare that they have no competing interests.

## Authors' contributions

MS was involved in the study designing, literature review, data collection, analysis and manuscript preparation. GS carried out the analysis and helped in the preparation of manuscript. KTH helped with data collection and was also involved in the analysis of the study. KAA was involved in studying comparing various pathological aspects of the specimen. ATS conceived of study, participated in its design and coordination, helped draft and revise the manuscript, and was responsible for surgical resection of all specimens. All authors read and approved the manuscript.
